# The Stethescope

**Published:** 1855-06

**Authors:** 


### THE STETHESCOPE.

THE STETHESCOPE, which has again changed hands, is said now to be placed on a permanent basis. GOODRIDGE A. WILSON, M. D., and RICHMOND A. LEWIS, M. D., its new editors, appear in the June number in a handsome salutatory, the conclusion of which is contained in the following extract:—"To the liberal minded—those who are in favor of the enactment of the reforms which the best heads and hearts of the profession have recommended—those who are proud of that profession, on account of its achievements in the past, and who would exalt its destiny in the future, and who would erect higher muniments of protection around its honor—to all such we appeal for their sympathy and counsel, and the contributions of their pens, in our efforts to conduct a fair, liberal and independent medical press."

The Stethescope is connected with no medical school, and has heretofore on various occasions, given some pretty hard blows to those institutions which being weighed in the balances used by its editors were found wanting. The tone of our Virginia confrére has always been high, and its course independent, albeit it has sometimes occurred to us that it was rather hasty at contradiction and too prone to apply the lash to those who did not wheel at the word of command into the ranks of the great army of Reform headed by that very notorious individual Young America. This was, we think, more observable during the administration of our friend Dr. GOOCH, the projector of the Stethescope, than when the journal was edited by the six gentlemen appointed by the State Medical Society. Under the direction of the latter corps, the Stethescope did good service in the cause of medical science and took rank among the leading medical periodicals of the country.

We wish Drs. WILSON and LEWIS abundant success in their enterprise. They assume the control of a journal which already has a large subscription list and what is of even more importance to its usefulness—with many valuable and industrious contributors. We trust they may continue to write up to their "platform" and reap a rich harvest both in honors and money.

D. W. Y.

BRANDENBERG, Meade Co., Ky.,

June 2, 1855.

*To the Editor of the Western Journal of Medicine & Surgery:*

DEAR SIR:—The friends of the Meade, Hardin, and Breckenridge County Medical Association, met at the Big Spring, on the 1st day of June, and organized by electing for one year the following officers:—J. G. Hicks, M. D., President; W. W. Board, M. D., Vice President; J. V. Withers, M. D., Secretary; H. K. Pusey, M. D., Cor. Secretary; F. H. McAtee, Treasurer; E. O. Brown, M. D., J. H. McCay, M. D., J. Odiorne, M. D., Executive Committee.

The next meeting of the Association will be held at Brandenburg, on the first Monday in December next. Letters should be addressed to the Cor. Secretary at Garnettsville, Meade Co., Ky.

J. V. WITHERS, *Secretary.*

We cheerfully give the above proceedings a place in our pages. We regret that there are not more such organizations in Kentucky. If conducted with the proper spirit they can but be productive of great good. Their sessions afford not only an opportunity for the interchange of opinions, the discussion of medical subjects and the like, but in a social point of view they are of signal benefit They bring the members of our common calling together—they diminish jealousies,—they allay sectional prejudices,—they correct error—they beget kindly feelings and make friends. We would suggest to the gentlemen composing the Association alluded to above that quarterly meetings would be better than meetings held only semi-annually. Two meetings during the course of the year are hardly sufficient.

